# Advancement of Gallium and Gallium-Based Compounds as Antimicrobial Agents

**DOI:** 10.3389/fbioe.2022.827960

**Published:** 2022-02-04

**Authors:** Fupeng Li, Fengxiang Liu, Kai Huang, Shengbing Yang

**Affiliations:** Shanghai Key Laboratory of Orthopaedic Implants, Department of Orthopaedic Surgery, Shanghai Ninth People’s Hospital, Shanghai Jiao Tong University School of Medicine, Shanghai, China

**Keywords:** gallium (Ga(III)), antibacterial, iron matabolism, optimization, bioavailability

## Abstract

With the abuse and misuse of antibiotics, antimicrobial resistance has become a challenging issue in the medical system. Iatrogenic and non-iatrogenic infections caused by multidrug-resistant (MDR) pathogens pose serious threats to global human life and health because the efficacy of traditional antibiotics has been greatly reduced and the resulting socio-economic burden has increased. It is important to find and develop non-antibiotic-dependent antibacterial strategies because the development of new antibiotics can hardly keep pace with the emergence of resistant bacteria. Gallium (III) is a multi-target antibacterial agent that has an excellent antibacterial activity, especially against MDR pathogens; thus, a gallium (III)-based treatment is expected to become a new antibacterial strategy. However, some limitations of gallium ions as antimicrobials still exist, including low bioavailability and explosive release. In recent years, with the development of nanomaterials and clathrates, the progress of manufacturing technology, and the emergence of synergistic antibacterial strategies, the antibacterial activities of gallium have greatly improved, and the scope of application in medical systems has expanded. This review summarizes the advancement of current optimization for these key factors. This review will enrich the knowledge about the efficiency and mechanism of various gallium-based antibacterial agents and provide strategies for the improvement of the antibacterial activity of gallium-based compounds.

## 1 Introduction

The emergence of drug-resistant bacteria has greatly reduced the therapeutic effect of traditional antibiotics, posing a great challenge to the global medical systems and requiring the research and development of novel non-antibiotic-dependent antibacterial strategies (Roope et al., 2019) to combat drug resistance. Gallium is a semi-metallic element whose citrate buffered nitrate form was approved by the United States Food and Drug Administration (FDA) for clinical use in the treatment of malignant tumor-related hypercalcemia and autoimmune diseases in 2003 (Leyland-Jones, 2003). Noteworthy, its antibacterial effect has gradually attracted attention in recent years.

In 1931, Levaditi et al. (1931) found that intravenous injection of gallium tartrate could eliminate both syphilis in rabbits and *Trypanosoma evansi* in mice. Moreover, Yukihiro et al. (Britigan et al., 2000; Kaneko et al., 2007; Lessa et al., 2012) found that gallium has a strong killing effect on *Pseudomonas aeruginosa*, a multi-drug resistant opportunistic pathogen that is also the main pathogenic bacterium responsible for respiratory failure in patients with pulmonary cystic fibrosis. The ideal bactericidal effect of gallium ions can be achieved even at micromolar concentration levels. In addition, gallium at such low concentrations can remove the biofilm formed by *P. aeruginosa*, which is difficult to eliminate by traditional antibiotics. Furthermore, Choi et al. (2017, 2019) demonstrated that gallium also has bactericidal effects against common drug-resistant bacteria, such as *Mycobacterium tuberculosis*, *Klebsiella pneumoniae*, and other Gram-negative bacteria.

Currently, it is believed that the mechanism underlying the gallium antibacterial effect is related to gallium uptake by bacteria as an iron mimic. Considering that gallium is a redox inert metal that cannot be reduced under physiological conditions, gallium-substituted enzymes cannot exert the functions of the corresponding iron-dependent enzymes, which are indispensable for bacterial proliferation; thus, iron metabolism is disturbed, leading to bacterial death (Kelson et al., 2013). Iron is an indispensable nutrient for bacterial proliferation, especially for bacteria that establish infections *in vivo*. Therefore, it is difficult for bacteria to evolve resistance to gallium by reducing uptake, because it would reduce iron uptake as well. Consequently, it is believed that gallium-based compounds may become the next generation of antibiotics because of their scavenging effect on resistant bacteria.

Although gallium has definite antibacterial effects, it is almost completely hydrolyzed into insoluble hydroxide under physiological conditions. Therefore, it is difficult to release adequate quantities of gallium (III) to play an antibacterial role due to its extremely low bioavailability (Bonchi et al., 2014). In contrast, the prevention and treatment of iatrogenic infections caused by implants or internal incisions requires the maintenance of antimicrobial agent concentrations above the minimum inhibitory concentration (MIC) at the infection foci, and the explosive release of an element in a short period of time would hardly meet the clinical needs (Zhang et al., 2015; Shao et al., 2016; Ning et al., 2018).

Besides, in contrast with the treatment of respiratory infections, implant-related anti-infective materials have to be implanted *in vivo* in the form of scaffolds, requiring good biomechanical properties and releasing antibacterial agents in a sustained manner. Finally, a combination of drugs with different antibacterial mechanisms could produce stronger synergistic antibacterial effects than a single antibacterial component; this has become a research hotspot. Various combinations of gallium ions and clinically applied antibiotics or other non-antibiotic antimicrobial agents are expected to potentiate the antibacterial effect of gallium (III) and enable the traditional antibiotics to be applied in the treatment of drug-resistant bacteria through synergistic effects (Garcia-Contreras et al., 2013). This review summarizes the above-mentioned methods for improving the antibacterial effect of gallium, explores the putative antibacterial mechanism of gallium, and provides ideas for the development of novel gallium-based antibacterial strategies and for promoting the clinical transformation of gallium-based drugs (Table 1).

**TABLE 1:** Summary of the use of gallium and gallium-compounds as antimicrobial agents.

Optimization strategies	Gallium and gallium-based compounds	Antimicrobial effects	References
coordination compound	gallium citrate, gallium maltolate, gallium tartrate, tris(8-quinolinolato) gallium (III) (KP46), and gallium (III) complexes of a-N-heterocyclic thiosemicarbazones	improved solubility and not poor bactericidal effects on drug-resistant Gram-negative and Gram-positive bacteria, including *P. aeruginosa*, *Acinetobacter baumannii*, *M. tuberculosis*, and methicillin-resistant *Staphylococcus aureus*	Bonchi et al. (2014), Piatek et al. (2020), Lessa et al. (2012)
	Ga_2_L_3_ (bpy)_2_, (L = 2,2′-bis(3-hydroxy-1,4naphthoquinone); bpy = 2,2′-bipyridine)	exert bactericidal effects on drug-resistant *P. aeruginosa* and *S. aureus* in an iron-containing environment	Wang et al. (2021b)
	GaMe_2_(L) and Ga (Me)_2_L	improved antibacterial activity than quinolinolate alone	Duffin et al. (2020)
nanomaterial-based vehicles	Lipo-Ga-GEN	improved antibacterial activity than the corresponding drugs without liposomes	Halwani et al. (2008)
	gallium-NAC	more gallium ions were deposited in *P. aeruginosa* cells in the gallium-NAC treatment group than in the traditional gallium citrate treatment group	Young et al. (2019)
	gallium-containing siderophores and heme analogues: desferoxamine-gallium, Ga-protoporphyrin IX, Ga-deuteroporphyrin, Ga-mesoporphyrin, Ga-hematoporphyrin, Ga-octaethylporphyrin, and Ga-porphine	not all siderophores combined with an antibacterial agent show increased antibacterial activity. Ga-protoporphyrin IX showed the best antibacterial effect	Kelson et al. (2013)
	ciprofloxacin-siderophore	decreased uptake of gallium and antibacterial potency compared to ciprofloxacin alone both in iron replete and deplete conditions	Sanderson et al. (2020)
	bioresponsive antibacterial nanomaterials based on gallium (III) and iron (III) cross-linked polysaccharide materials	improved bioavailability of gallium	Lin et al. (2021), Best et al. (2020)
alloys and scaffold composites	Ga-doped titanium alloys	long-lasting release of Ga (III) and strong antibacterial effects on multidrug-resistant *S. aureus* for at least 3 days	Cochis et al. (2019)
	Ga-doped magnesium alloys	effective in the treatment of osteomyelitis	Gao et al. (2019)
	eutectic gallium–indium alloys	time-increasing bactericidal effects against Gram-positive bacteria	Li et al. (2021b)
	bioglasses doped with gallium	sustained release of gallium ions and time-increasing bactericidal effects	Kurtuldu et al. (2021), Mehrabi et al. (2020), Wang et al. (2021a), Siqueira et al. (2019), Song et al. (2019), Lapa et al. (2019), Ciraldo et al. (2021), Keenan et al. (2017), Rahimnejad Yazdi et al. (2018)
	gallium-doped zinc borate bioactive glass	sustained and controlled release of gallium for at least 28 days	Rahimnejad Yazdi et al. (2018)
	phosphate glass, hydroxyapatite, PCL and hydrogel, collagen, poly (4-hydroxybutyrate), silk fibroin, Ca titanate	sustained release of gallium ions and play an excellent bactericidal effect against common pathogens, such as *E. coli*, *S. aureus*, and *P. aeruginosa*, both *in vivo* and *in vitro*	Lapa et al. (2020), Pajor et al. (2020), Rastin et al. (2021), Xu et al. (2019), Muller et al. (2021), Mehrabi et al. (2020), Rodriguez-Contreras et al. (2020)
layered double hydroxide	gallium (Ga)–strontium (Sr) layered double hydroxides	sustained release of Ga ions and time-increasing bactericidal effects	Li et al. (2021a)
	gallium (Ga)–zinc (Zn) layered double hydroxides	sustained release of Ga ions and time-increasing bactericidal effects	Donnadio et al. (2021)
Synergistic strategies	ciprofloxacin, colistin, meropenem, and tobramycin	restored the bactericidal effect of traditional antibiotics and reversed the drug resistance of resistant bacteria	Rezzoagli et al. (2020)
	tetracycline	improved antibacterial activity of gallium nitrate both *in vitro* and *in vivo*	Kang et al. (2021)
	poly (ethylene glycol)-desferrioxamine/gallium (PEG-DG) conjugates	Increase bacterial susceptibility to vancomycin	Qiao et al. (2021a)
	a xenosiderophore-conjugated cationic random copolymer	0.31 of FICI for *P. aeruginosa*	Qiao et al. (2021b)
	a gallium-chitosan complex	improved antibacterial activity than that of single chitosan	Akhtar et al. (2020)
	ciprofloxacin-functionalized desferrichrome	improved antibacterial activity than that of ciprofloxacin alone	Pandey et al. (2019)
	metal ions	silver ions, zinc ions, Cd, Se, and Ga had good synergistic effects	Aziz (2019), Pormohammad and Turner (2020); Vaidya et al. (2019), Yoon et al. (2006)
	gallium-substituted hemoglobin combined with Ag nanoparticles	improved antibacterial activity	Morales-de-Echegaray et al. (2020)
	gallium-porphyrin, gallium-substituted hemoglobin, phthalocyanine, indocyanine green (ICG), hollow titanium dioxide nanotubes and gallium ions	improved antibacterial activity	Morales-de-Echegaray et al. (2020); Shisaka et al. (2019), Xie et al. (2021), Rahimnejad Yazdi et al. (2018)
	nitrates and gallium ions	induce antibacterial activity against *P. aeruginosa* under both aerobic and anaerobic conditions	Zemke et al. (2020)
	graphene foam and gallium ions	improved antibacterial activity	Slate et al. (2021)

## 2 Biochemical Properties of Gallium and Iron Ions

Gallium is an element of group IIIA of the periodic table, with an atomic number of 31 and a molecular weight of 69.72 g/mol. The chemical behavior of gallium is close to that of Fe(III) in terms of its electrical charge, ion diameter, coordination number, electron affinity, tendency to form ionic bonds, ionization potential, and electron configuration. The exact parameters are summarized in Table 2. Another similarity between these elements is the extensive hydrolysis that occurs in neutral aqueous solutions, with the consequent formation of various hydroxide species, such as Ga(OH)_4_ and Ga(OH)_3_, for that Ga(III) ions, having low polarization and a small size, are hard acids with high affinity for hydroxide ions (Nielsen et al., 2021). Although gallium hydroxide precipitates under physiological conditions, it is soluble under acidic or alkaline conditions. When the pH of the solution changes from low to high, gallium hydroxide changes from a dissolved state to a precipitated state in the form of Ga(OH)_3_ and then back to a dissolved state as Ga(OH)_4_. However, it is worth noting that gallium is a redox inert element that cannot be reduced to Ga(II) under physiological conditions (Centola et al., 2020).

**TABLE 2:** Similarities between gallium and iron ions.

	Gallium (III)	Iron (III)
Octahedral ion radius	0.620 Å	0.645 Å
Tetrahedral ion radius	0.47 Å	0.49 Å
Ionization potential	64 eV	54.8 eV
Ionization affinity	30.71 eV	30.65 eV

The proteins responsible for iron transport *in vivo*, such as transferrin and lactoferrin, can also bind stably with gallium. However, gallium-transporter complexes are more sensitive to acidic pH than iron-transporter complexes, and strong dissociation of gallium ions can occur at pH 6.1 (Bonchi et al., 2014).

## 3 Antibacterial Mechanism of Gallium Ions

### 3.1 Iron Uptake

**FIGURE 1:**
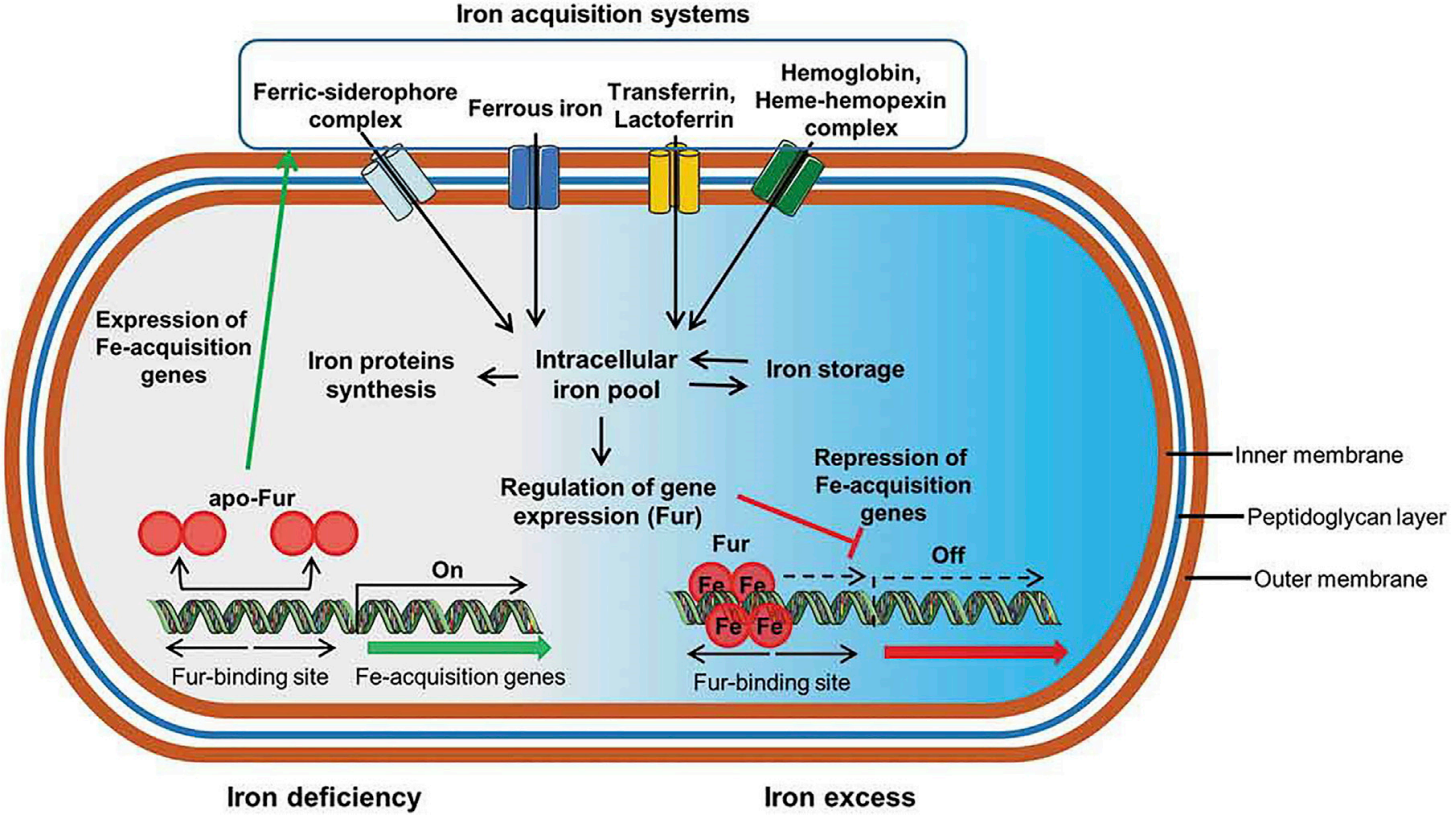
Schematic representation of critical iron acquisition mechanisms and pathways in bacteria. Both Gram-negative and Gram-positive bacteria have evolved multiple sophisticated systems to acquire iron from the environment, including trivalent iron containing siderophores, hemes, heme proteins, heme analogues, and transferrin/lactoferrin. Due to the similarity between gallium and iron, the above pathways are also suitable for gallium transport, providing the possibility of targeted therapy for gallium (Seyoum et al., 2021).

Iron is an essential nutrient for the survival and proliferation of both bacteria and cells; it is a universal cofactor of oxidoreductases that participates in a variety of critical metabolic pathways *in vivo*, such as DNA synthesis, electron transfer, and anti-oxidative stress (Andrews et al., 2003). As a result, bacteria that colonize a host and establish an infection require more iron than free living bacteria to maintain their fundamental nutrient needs for growth. Due to the low bioavailability of iron in the body, bacteria have evolved a complicated iron uptake mechanism, whereby continuous evolution and mutation, to compete with cells and other microorganisms in the body for iron to survive and reproduce. At present, most studies believe that bacteria mainly obtain iron from the outside world in three ways (Figure 1): 1) siderophore-based systems, 2) heme/hemeprotein-based systems, and 3) transferrin and lactoferrin-based systems (Kelson et al., 2013).

Siderophores (Wilson et al., 2016) are small molecules secreted by microorganisms; siderophores can specifically recognize and bind to ferric ions from the host or other exogenous sources. In addition, bacteria simultaneously secrete heme carriers to capture hemes and heme analogues in the host. Heme is an important prosthetic group of human heme proteins, such as hemoglobin and myoglobin, which are responsible for oxygen transport. Kelson et al. (2013) systematically summarized the siderophores and heme carriers secreted by common pathogens. For example, pyoverdine and pyochelin, two types of siderophores secreted by *P. aeruginosa*, are critical for iron acquisition by this bacterium.

Bacteria can acquire iron depending on siderophores or heme carriers, but bacteria can also directly interact with iron sources, such as iron or hemes, and transport them into the cell through membrane receptors. Gram-positive bacteria specifically recognize the surrounding iron or iron-containing siderophores and hemes through receptors on the cytoplasmic membrane and then transport them to the cytoplasm where trivalent iron is dissociated from the complex and reduced to divalent iron for further use. In Gram-negative bacteria, the iron source is first recognized by an outer membrane receptor and then transported into the periplasm where it may enter the cell through the plasma membrane.

### 3.2 Iron Contender

Owing to the extremely similar biochemical characteristics of gallium and iron, it is difficult for biological systems to distinguish between gallium (III) and iron (III) and their corresponding compounds, such as iron protoporphyrin and gallium protoporphyrin; this is also the basis for the specific uptake of gallium by bacteria according to the above-mentioned pathways. Therefore, gallium can be used as an antagonist of iron to competitively bind to iron-dependent proteins, thus affecting the function and activity of related enzymes. In summary, gallium could be firmly bound to the siderophores that should be combined with iron, reducing the amount of iron obtained, and gallium complexes enter the bacterial cells as Trojan horses, but cannot undergo redox, interrupting the normal enzyme metabolism.

### 3.3 Disturbance of Iron Metabolism

Considering that gallium (III) cannot be reduced to divalent gallium under physiological conditions, when an enzyme that regularly has iron as a cofactor replaces it for gallium, its activity is affected, and its related biochemical processes are interrupted. It is believed that the inhibition of bacterial activity caused by iron metabolism disorders is mainly due to the inactivation of deoxynucleotide reductase. However, other important factors include the electron transfer, Krebs cycle, and protein synthesis. Ribonucleotide reductase (Kircheva & Dudev, 2021) is an indispensable iron-containing enzyme that participates in DNA replication and is essential for bacterial proliferation.

Recent studies have shown that oxidative stress caused by iron metabolism disorders plays a pivotal role in the antibacterial effect of gallium. Interestingly, it has been shown that the overexpression of NADPH-producing enzymes can counteract the oxidative stress evoked by gallium (Beriault et al., 2007). Zeng et al. (2021) also found that *Escherichia coli* strains with mutations in the *evgS* gene, encoding ROS detoxification enzymes, developed tolerance to gallium nitrate. Besides, the antibacterial effect of gallium ions on aerobic bacteria is better than that on anaerobic bacteria, which also indicates that reactive oxygen may be involved in the antibacterial mechanism of gallium (Zemke et al., 2020). The molecular mechanism of the antibacterial action of gallium ions remains to be further explored.

In addition to inhibiting bacterial proliferation by disturbing the activity of iron-containing enzymes such as deoxyribonucleotide reductase, gallium ions can reduce the production of virulence factors. Among virulence factors, biofilm formation is a crucial mechanism for multidrug resistance (Zhang et al., 2021). Bacteria secrete extracellular polymers to protect them from external antibiotics and the host immune system. In addition, the phenotypes of bacteria inhabiting biofilms are altered compared to that of planktonic bacteria. The proliferation rate tends to be lower, and the sensitivity to antibiotics is significantly reduced. Kang and Kirienko (2018) demonstrated that iron is indispensable for the formation of biofilms. Inactivation of pyoverdine, a key siderophore for *P. aeruginosa*, prevents biofilm formation; however, supplementation with ferric citrate can restore this ability. Therefore, gallium, as an iron antagonist, can affect the virulence of bacteria by reducing the iron content. A proteomic analysis suggested that gallium ions could reduce the expression abundance of quorum-sensing and swarming motility related proteins in *P. aeruginosa*; these proteins play a fundamental role in bacterial virulence and dissemination (Piatek et al., 2020).

## 4 Optimization of Gallium-Based Compounds

### 4.1 Development of Gallium-Based Clathrates

Gallium ions are highly hydrolyzable under physiological conditions, and trivalent gallium ions are almost completely hydrolyzed into insoluble hydroxides in the body, leading to extremely low bioavailability. Consequently, there are few gallium ions entering bacterial cells *in vivo* to play an antibacterial role. At present, the problem of precipitation caused by the hydrolysis of gallium is mainly solved by chelating various organic ligands with gallium to form complexes with increased water solubility. The precipitation of hydroxides may be prevented by surrounding the gallium cation with an appropriate ligand sphere, rendering it stable to hydrolysis. Furthermore, the pharmacokinetics of gallium-based drugs is closely related to their coordination chemistry, and different ligands have a profound impact on the water solubility of gallium-based drugs. Currently, mature gallium salts and gallium-based coordination compounds, such as gallium nitrate, gallium chloride, gallium citrate, gallium maltolate, gallium tartrate, tris(8-quinolinolato) gallium (III) (KP46), and gallium (III) complexes of a-N-heterocyclic thiosemicarbazones, with improved solubility and not poor bactericidal effects on drug-resistant Gram-negative and Gram-positive bacteria, including *P. aeruginosa*, *Acinetobacter baumannii*, *M. tuberculosis,* and methicillin-resistant *Staphylococcus aureus*, are being tested.

**FIGURE 2:**
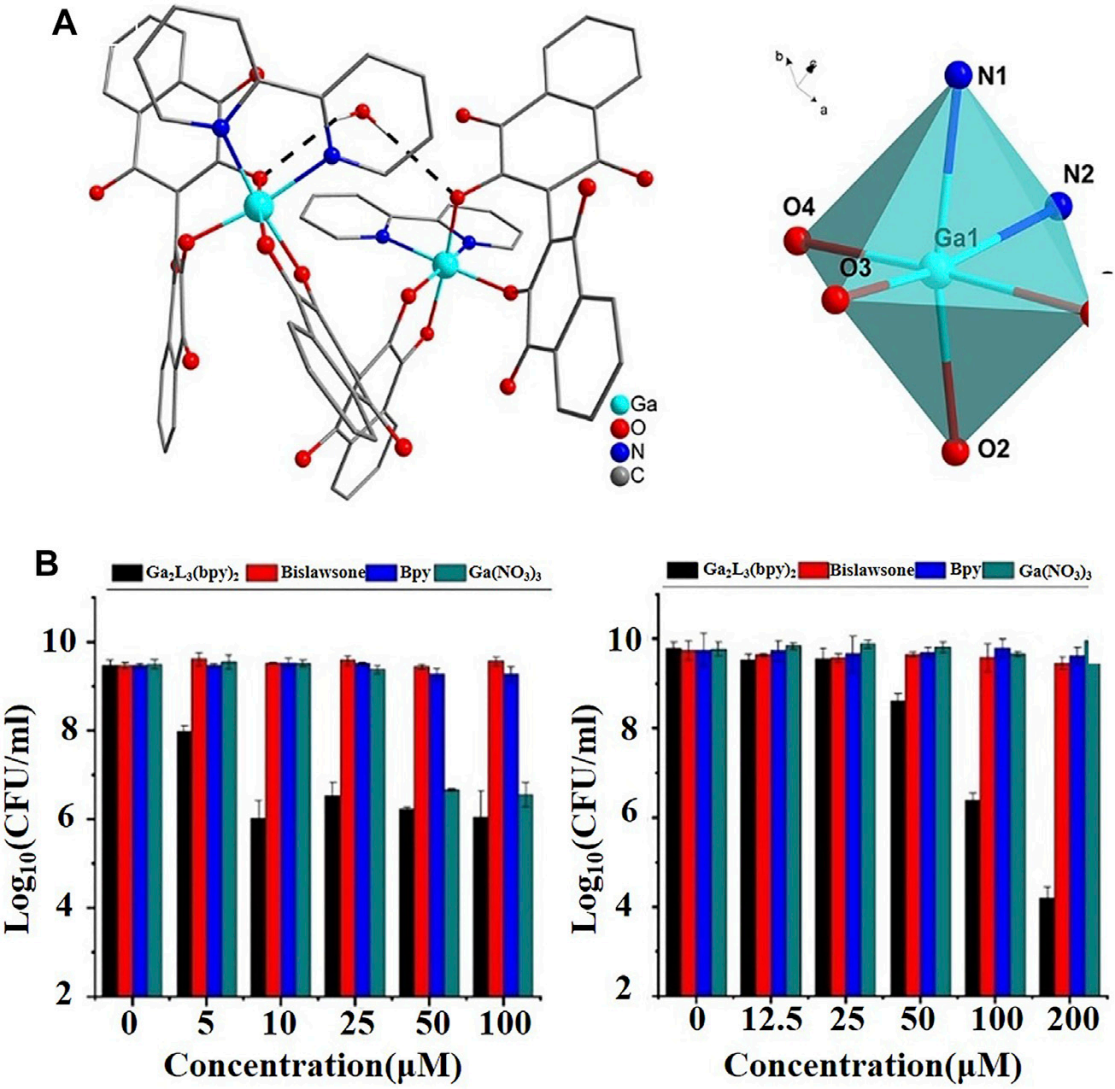
**(A)** Molecular structure of [Ga_2_L_3_(bpy)_2_]·H2O as determined by single-crystal X-ray structure analysis (left), and schematic representation of the coordination environment of the Ga(III) ion (right) (Wang et al., 2021b). **(B)** Antibacterial effect of [Ga_2_L_3_(bpy)_2_] on drug-sensitive *P. aeruginosa* ATCC 15692 (left) and drug-sensitive *S. aureus* ATCC 6538 (right) compared with those of gallium nitrate, bpy, and bislawsone (Wang et al., 2021b).

Bonchi et al. (2014) systematically summarized the pharmacological properties and antibacterial activities of the gallium salts and gallium-based complexes mentioned above. Among them, gallium maltoate, as a second-generation gallium-based coordinated complex, may be used in an oral or topical way with obviously improved bioavailability of gallium ions compared to that of gallium nitrate. Piatek et al. (2020), using quantitative proteomics, further revealed that gallium maltoate exerted antibacterial effects by inhibiting the quorum-sensing system in *P. aeruginosa*. Similar to gallium maltoate, KP46 was also designed to improve the oral bioavailability of gallium ions. Thiosemicarbazones, antibacterial and antitumor drugs, can also be applied as ligands to form complexes with gallium ions (Enyedy et al., 2012). Lessa et al. (2012) demonstrated that gallium-based compounds coordinated with 2-formylpyridine-, 2-acetylpyridine-, and 2-benzoylpyridine-derived thiosemicarbazones not only potentiated the antibacterial activity of gallium ions due to their increased bioavailability, but also improved the antibacterial activity and spectrum of thiosemicarbazones. New gallium complexes have also been developed with the continuous discovery of ligands. Wang et al. (2021b) synthesized a lipophilic ligand, Ga_2_L_3_(bpy)_2_, (L = 2,2′-bis(3-hydroxy-1,4naphthoquinone); bpy = 2,2′-bipyridine) (Figure 2A), which had excellent bactericidal effects on drug-resistant *P. aeruginosa* and *S. aureus* in an iron-containing environment, with MIC of 10 and 100 μM, respectively (Figure 2B). The antibacterial effect was superior to that of gallium alone. More interestingly, the lipophilic gallium ligand delayed the emergence of bacterial resistance. Duffin et al. (2020) synthesized a series of eight alkyl gallium complexes of general formulae [GaMe_2_(L)] and [Ga(Me)_2_L] to increase the release of gallium and promote a reaction with transferrin and lactoferrin. The antibacterial activity of alkyl gallium (III) quinolinolate was three times that of quinolinolate alone. It is worth noting that the category and quantity of alkyl gallium (III) quinolinolate had a certain influence on the lipid solubility of the complex, providing a reference for the development of new antibacterial gallium-based complexes. In addition, gallium-based complexes with tranexamic acid and pyrophosphate as ligands have achieved great success as antitumor agents, given the similarity of bacterial and tumor metabolisms (El-Habeeb and Refat, 2019). Their application in the field of antibacterial therapy is worthy of further study (Greenfield et al., 2019).

### 4.2 Advancement in the Development of Nanomaterial-Based Vehicles for Loading Gallium Ions

The emergence of diverse nanomaterials provides an excellent platform for loading gallium ions. On the one hand, the ideal nanomaterial surface-capping agent could improve the biocompatibility of gallium ions and increase the therapeutic window range; on the other hand, it could effectively reduce the hydrolysis of gallium (III), thus, releasing more gallium ions at the infection site. Halwani et al. (2008) used liposomes as carriers to encapsulate and transport gallium ions and improve their bioavailability by taking advantage of the lipophilic characteristics of liposomes. The complexes demonstrated excellent antimicrobial activity against *P. aeruginosa* and the corresponding biofilms compared to Ga(NO_3_)_3_ alone. Lipo-Ga-GEN, liposomes carrying both gallium ions and gentamicin (0.6 µM for Ga and 8 mg/L for GEN, respectively), inhibited bacterial growth completely, while other formulations failed to achieve the same effect at the same drug concentrations. In addition, Lipo-Ga-GEN, Lipo-Ga (liposomes carrying gallium ions only), and Lipo-GEN (liposomes carrying gentamicin only) exhibited better antibacterial activities than the corresponding drugs without liposomes (Halwani et al., 2008), providing another way to improve the bioavailability of gallium. Similar to liposomes, N-acetyl-cysteine (NAC), a thiol-substituted derivative of the amino acid l-cysteine, has been used as a surface-capping agent for gallium because of its excellent biocompatibility and water solubility (Young et al., 2019). The amount of gallium ions released by the NAC-coated gallium particles was much higher than that of the gallium particles without NAC coating. Noteworthy, more gallium ions were deposited in *P. aeruginosa* cells in the gallium-NAC treatment group than in the traditional gallium citrate treatment group, indicating that NAC promoted the absorption of gallium ions.

In addition, the construction of gallium-containing siderophores and heme analogues, taking advantage of the iron uptake pathway, provides another strategy for the preparation of gallium-loaded nanomaterials. The targeted accumulation of gallium in bacterial cells depends mainly on the formation of couplings with endogenous siderophores or hemes. Through the Trojan horse strategy, gallium is transported into bacterial cells with the help of the bacteria’s own iron acquisition receptors. The antibacterial activities of common gallium-siderophores and gallium-heme conjugates have been summarized by Kelson et al. (2013), including those of desferoxamine-gallium, Ga-protoporphyrin IX, Ga-deuteroporphyrin, Ga-mesoporphyrin, Ga-hematoporphyrin, Ga-octaethylporphyrin, and Ga-porphine. Among them, Ga-protoporphyrin IX, as a heme analogue, showed the best antibacterial effect and had a good bactericidal effect on both Gram-positive and Gram-negative bacteria for perturbing the metabolism of heme. It is worth noting that not all siderophores combined with an antibacterial agent show increased antibacterial activity (Frangipani et al., 2014).

**FIGURE 3:**
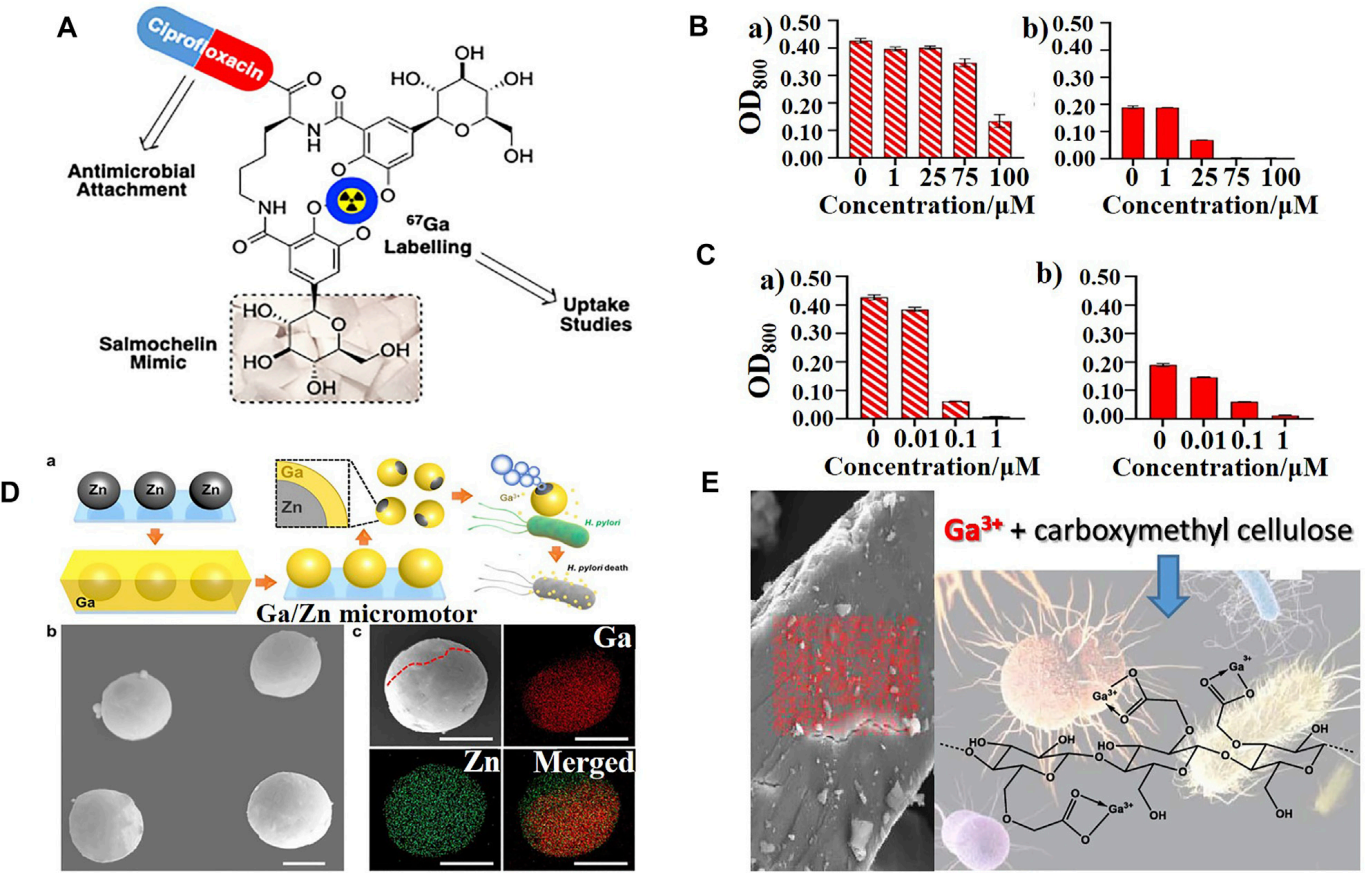
**(A)** Schematic representation of a ciprofloxacin−siderophore Trojan horse antimicrobial (Sanderson et al., 2020). **(B,C)** Growth status of *E. coli* K12 (BW25113) in the presence of **(B)** ciprofloxacin−siderophores and **(C)** ciprofloxacin after 48 h with **(a)** sufficient iron levels or **(b)** scarce iron (Sanderson et al., 2020). **(D)** Schematic representation of the synthesis and antibacterial mechanism of Janus Ga/Zn micromotors and their characterization (Lin et al., 2021). **(E)** Scheme of gallium molecules encapsulated by carboxymethyl cellulose (CMC) (Best et al., 2020).

Sanderson et al. (2020) synthesized a novel ciprofloxacin-siderophore antimicrobial (Figure 3A) by incorporating key design features of salmochelin, a stealth siderophore expressed by many Enterobacteriaceae; however, it decreased the uptake of gallium and antibacterial potency compared to ciprofloxacin alone both in iron replete and deplete conditions (Figures 3B,C). Frangipani et al. (2014) compared the antibacterial activity of several commonly used siderophore-gallium complexes with gallium nitrate alone, and the results were similar to those described above. Only the complex formed with the endogenous siderophore, the pyochelin-gallium complex, significantly potentiated the antibacterial activity of gallium ions, whereas those with ferrichrome, desferrioxamine, and pyoverdine, alleviated the antibacterial effect of gallium nitrate. These results suggest that the type of siderophore and the size and polar surface area of the corresponding conjugate remain significant challenges in the design of Trojan horse antimicrobials, which may prevent gallium from being recognized by receptors of the outer membrane.

In addition to gallium-ligand and gallium-iron/heme carriers, Lin et al. (2021) provided a new strategy: the active uptake of gallium by bubble-propelled Janus gallium/zinc micromotors that, at an acidic microenvironment, can increase the gallium content in bacteria (Figure 3D). Based on the same principle, Best et al. (2020) prepared a family of bioresponsive antibacterial nanomaterials based on gallium (III) and iron (III) cross-linked polysaccharide materials (Figure 3E), to which *P. aeruginosa* was specifically responsive, to improve the bioavailability of gallium ions.

### 4.3 Construction of Gallium-Doped Alloys and Scaffold Composites

Although gallium has a definite antibacterial effect, existing gallium-based drugs, including gallium nitrate, tend to quickly release gallium (III) *in vivo*, reaching the maximum concentration in an extremely short time and failing to exert an antibacterial effect. Opportunities for iatrogenic infections exist anytime, and it is particularly critical that the concentration of an antibacterial agent at infective sites is maintained above the MIC. The controlled and sustained release of antibacterial agents is of great significance for the prevention and treatment of clinically common implant-related infections and skin incision-related infections. In addition, gallium salts and gallium-based complexes are mostly administered intravenously or orally, while local treatments tend to be safer, which is of great clinical significance for local infection control.

**FIGURE 4:**
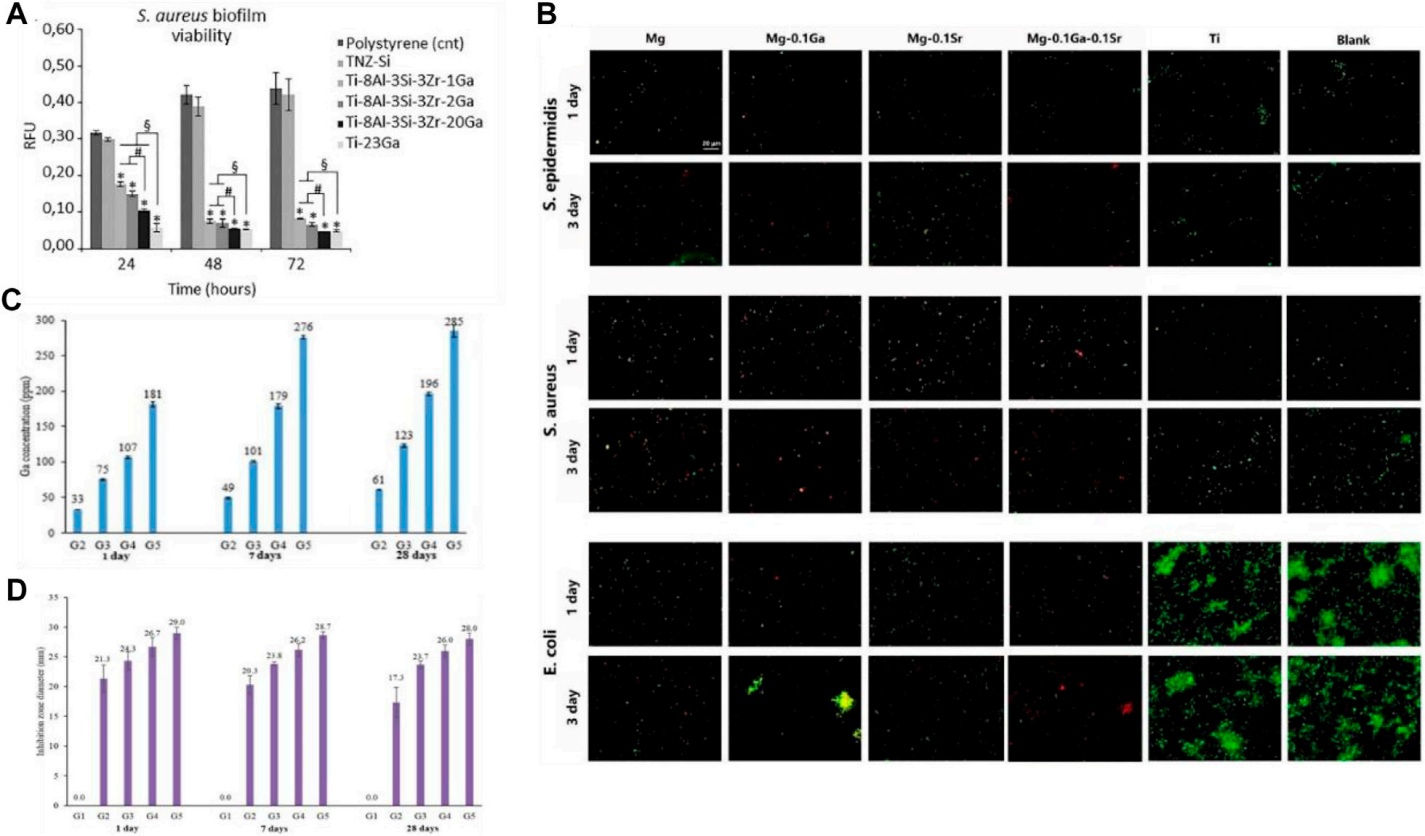
**(A)** Antibacterial effect of metallurgical gallium additions to titanium alloys against *S. aureus* biofilm formation (Cochis et al., 2019). **(B)** Fluorescent images of biofilms formed by *Staphylococcus epidermidis*, *S. aureus*, and *E. coli* on pure Mg, Mg-0.1 Ga, Mg-0.1 Sr, Mg-0.1 Ga-0.1 Sr, and c.p. Ti surfaces at days 1 and 3; biofilms were detected through live-dead staining (Gao et al., 2019). **(C)** Accumulated concentration of gallium ions released from different borate glasses with increased gallium content (0, 2.5, 5, 10, and 15 Wt % Ga) after being immersed in deionized water for 1, 7, and 28 days at 37°C (Rahimnejad Yazdi et al., 2018). **(D)** Evaluation of long-time antibacterial activity of the borate glasses against *P. aeruginosa* after 1, 7, and 28 days of incubation; inhibition was determined by measuring inhibition zone diameters (Rahimnejad Yazdi et al., 2018).

Innovations in biomaterials, such as hydrogels and bioglasses, as well as improvements in manufacturing processes, such as coating technology, 3D printing technology, and metallurgy technology, make it possible for gallium-loaded scaffolds and alloys to be applied in the body as tissue engineering materials. In view of the low melting point (15.5°C) of gallium and its similarity with aluminum, it is possible to microalloy gallium with other biocompatible metals. Cochis et al. (2019) investigated Ga-doped titanium alloys using metallurgical methods, and the results demonstrated that these alloys ensured long-lasting release of Ga (III) and strong antibacterial effects on multidrug-resistant *S. aureus* for at least 3 days, showing a high potential for the treatment of implant-related infections in orthopedics. After metallurgical addition of gallium, the antibacterial activity of titanium alloys was significantly improved compared with that of polystyrene, and the antibacterial activity could still be observed after 3 days. Although the antibacterial activity over longer periods of time was not studied, the effect would be expected to be good after observing the trend. In addition, with the increase in gallium content, the antibacterial activity of the alloy gradually improved; however, the mechanical properties of the titanium alloy might also be affected, making it necessary to further explore the optimal gallium loading amount (Figure 4A).

Similarly, through gallium-strontium microalloying, magnesium alloys (Gao et al., 2019) have also been shown to be effective in the treatment of osteomyelitis. Compared with titanium, magnesium is degradable under physiological conditions with certain advantages in the treatment of bone infections; moreover, magnesium allows for the sustained release of gallium (III). According to the observations from fluorescent imaging studies (Figure 4B), magnesium alloys combined with gallium almost eliminated *S. aureus* and *E. coli* after 3 days. The red fluorescence intensity of the Ga-containing magnesium alloy increased gradually and reached a maximum on the third day, indicating the slow-release effect of gallium ions. Besides, 3D printed gallium-based liquid metals such as eutectic gallium–indium alloys have also shown time-increasing bactericidal effects against Gram-positive bacteria (Li K. et al., 2021).

With the development of manufacturing technology and the constant updating of biological materials, an increasing number of materials are used as scaffolds to store antibacterial agents to achieve a slow release. Mesoporous bioactive glasses (Kurtuldu et al., 2021) have been widely applied *in vivo* in non-weight-bearing parts of the body such as the oral cavity, in orthopedics, and in skin tissues where they act as repair materials and drug-loading scaffolds (Mehrabi et al., 2020). Recently, bioglasses doped with gallium have been gradually used as antibacterial materials against bone defect infections (Wang et al., 2021a), caries infections (Siqueira et al., 2019; Song et al., 2019), and skin incision infections (Lapa et al., 2019). The *in vitro* ion release curves from these gallium-loaded complexes show that gallium can be continuously released for nearly 550 h and that the concentration of gallium ions can reach 20 ppm on day 21 (Ciraldo et al., 2021), which may be attributed to the network structure and composition of the bioglass, especially to its calcium content (Keenan et al., 2017; Rahimnejad Yazdi et al., 2018).

Rahimnejad Yazdi et al. (2018) synthesized a gallium-doped zinc borate bioactive glass exhibiting a sustained and controlled release of gallium for at least 28 days. Interestingly, different gallium contents resulted in different gallium ion release curves (Figure 4C) and different antibacterial effects (Figure 4D). The cumulative concentrations of gallium (III) in all gallium-loaded bioglasses under a simulated body fluid environment increased gradually, and the highest gallium content reached 285 ppm on day 28. The controlled release of gallium ions was consistent with the sustained bacteriostatic activity against *P. aeruginosa* over 28 days. Other biomaterials, including phosphate glass (Lapa et al., 2020), hydroxyapatite (Pajor et al., 2020), PCL and hydrogel (Rastin et al., 2021), collagen (Xu et al., 2019), poly (4-hydroxybutyrate) (Muller et al., 2021), silk fibroin (Mehrabi et al., 2020), and Ca titanate (Rodriguez-Contreras et al., 2020), have also been proven to compound with gallium for a sustained release of gallium ions and to play an excellent bactericidal effect against common pathogens, such as *E. coli*, *S. aureus*, and *P. aeruginosa*, both *in vivo* and *in vitro*.

The successful synthesis of gallium-sustained release materials is of great clinical significance. On the one hand, hydrogel, bioceramics, and other materials with good biocompatibility can be directly used as tissue engineering scaffolds for the repair of infectious defects. On the other hand, chemical processes, such as electrophoretic deposition, thermochemical treatment (Rodriguez-Contreras et al., 2020), solid-state method (Pajor et al., 2020), radio-frequency magnetron sputtering (Stan et al., 2020), and gel/sol can form antibacterial coatings (Pajor et al., 2020; Rodriguez-Contreras et al., 2020; Stan et al., 2020; Almohandes et al., 2021; Centurion et al., 2021) containing gallium on the surface of implants to prevent and treat iatrogenic infections. The controlled release of gallium by the above materials mainly benefits from their biodegradability, porous network structure, and adjustable porosity.

### 4.4 Design of Gallium-Containing Layered Double Hydroxide

**FIGURE 5:**
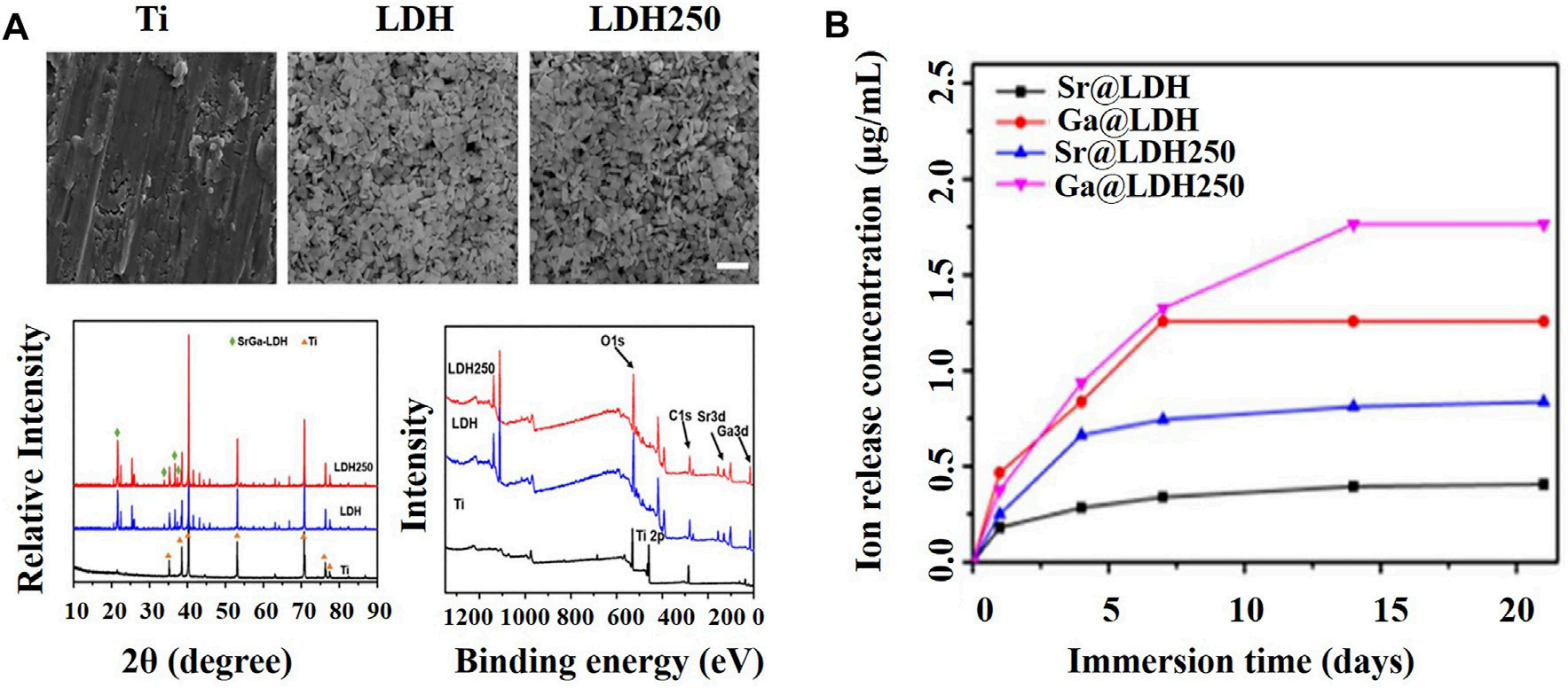
**(A)** Characterization of Ti, LDH, and LDH250 substrates (Li K. et al., 2021). **(B)** The cumulative release of gallium and strontium ions from the layered double hydroxide (LDH) and layered double hydroxide calcined at 250°C (LDH 250) was observed within 20 days of soaking in PBS solution (Li K. et al., 2021).

LDHs are layered solids described by the general formula [M(II)_1−x_M(III)_x_ (OH)_2_](A^n−^)_x/n_·nH2O, where M(II) is a divalent cation, M(III) is a trivalent cation, and A^n−^ represents the anions that balance the positive charges of the lamellae. LDHs have attracted considerable attention as drug release platforms and bone tissue engineering materials based on their excellent biocompatibility (Taviot-Guého et al., 2018). The region between layers can store a large amount of bioactive ions where drugs are released in a sustained manner. In addition, LDHs can also be combined with other materials such as hydroxyapatite without affecting their own structure (Donnadio et al., 2021), which is beneficial for realizing the multi-functionalization of gallium-containing materials. Li K. et al. (2021) and Donnadio et al. (2021) constructed and characterized gallium (Ga)–strontium (Sr) layered double hydroxides (Figure 5A) and gallium (Ga)–zinc (Zn) layered double hydroxides, respectively, both of which exhibited sustained release of Ga ions. On day 21, the concentrations of Ga (III) released from LDH 250 and LDH reached almost 1.75 and 1.25 μg/ml, respectively (Figure 5B).

### 4.5 Synergistic Antimicrobial Effects Between Gallium and Other Antibacterial Agents

Recently, the combination of multiple antibacterial agents to achieve enhanced antibacterial effects has become a research hotspot (Pormohammad & Turner, 2020). With the excessive use of antibiotics and metal ions, bacteria gradually acquire drug resistance through horizontal gene transmission or gene mutation (Sutterlin et al., 2017). Some researchers have isolated bacterial strains that are resistant to gallium ions (Garcia-Contreras et al., 2013). The combination of two or more drugs with different antimicrobial mechanisms may reduce or delay the emergence of antimicrobial resistance by attacking different bacterial targets (Cottarel & Wierzbowski, 2007; Fischbach, 2011). In addition, the combined use of multiple antibacterial agents can reduce the therapeutic dose and concentration of a single antibacterial agent, thus reducing toxicity and side reactions, increasing the treatment window, and improving biological safety (Harrison et al., 2008).

The diversity of existing antibacterial agents has promoted a diversity of combinations with gallium ions. Among them, the combination of traditional antibiotics and gallium-based antimicrobials has attracted considerable attention. Rezzoagli et al. (2020) evaluated the synergistic antibacterial effect of gallium ions in combination with four clinically common antibiotics (ciprofloxacin, colistin, meropenem, and tobramycin) using a 9 × 9 drug concentration matrix. The results showed that gallium ions not only restored the bactericidal effect of traditional antibiotics, but also reversed the drug resistance of resistant bacteria. A research conducted by Kang et al. (2021) demonstrated that tetracycline could inhibit the biosynthesis of the endogenous siderophore pyoverdine at concentrations lower than the MIC, thus enhancing the antibacterial activity of gallium nitrate both *in vitro* and *in vivo*. In other words, gallium ions could reduce the concentration of conventional antibiotics for treatment, allowing antibiotics to play a role in the treatment of resistant bacteria. In addition to gallium nitrate, the Ga(III)-based compounds mentioned above, including Ga-siderophores and Ga-porphyrin, were used as adjuvants of antibiotics for antibacterial purposes. Both gallium-porphyrin and gallium-heme targeted the bacterial iron metabolism pathway because they were specifically and directly recognized and absorbed by bacterial membrane receptors. Therefore, gallium complexes bound to antibiotics entered bacterial cells, increasing antibiotic sensitivity, and playing a synergistic role. For example, protoporphyrin IX, which serves as a heme analogue, has been demonstrated to have enhanced antibacterial activity against several bacterial species by targeting cytochromes and interfering with cellular respiration (Hijazi et al., 2017).

**FIGURE 6:**
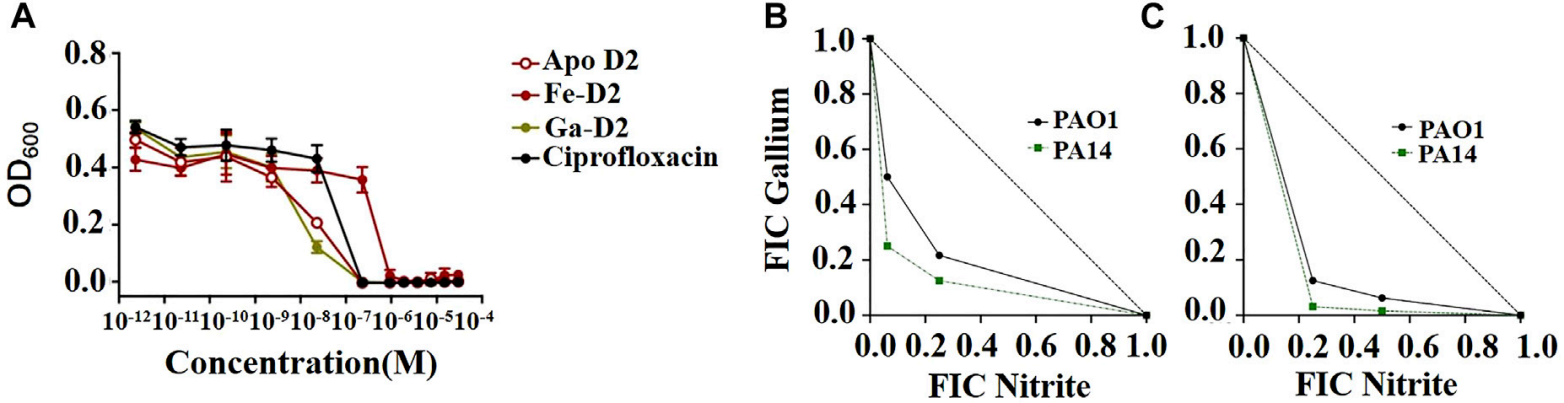
**(A)** MIC assays using a gallium-based complexes of ciprofloxacin-functionalized desferrichrome (D2) against *E. coli* K12 indicated that the Ga-D2 complex has a greater antibacterial potential when compared to that of ciprofloxacin alone (Pandey et al., 2019). **(B,C)** Synergistic antibacterial activity between nitrite and Ga^3+^. The isobolograms show the results of checkerboard assays for *P. aeruginosa* PAO1 and PA14 showing the fractional inhibitory concentration (FICs) of the two compounds in combination under **(B)** aerobic and **(C)** anaerobic conditions (Zemke et al., 2020).

Pandey et al. (2019) demonstrated that ciprofloxacin-functionalized desferrichrome exhibited a MIC of 0.23 μM for *E. coli*, in accordance with its parent fluoroquinolone antibiotic, and showed potent against *P. aeruginosa*, *S. aureus*, and *K. pneumoniae* with MICs of 3.8, 0.94, and 12.5 μM, respectively. The dose-response curve showed that the antibacterial effect of ciprofloxacin-functionalized gallium- desferrichrome conjugates was superior to that of ciprofloxacin alone (Figure 6A). In addition to using antibiotics directly, Qiao et al. (2021a) introduced PEG to further increase bacterial susceptibility to vancomycin, demonstrating excellent antibacterial activity.

Considering the excellent synergistic antibacterial effects of traditional antibiotics and gallium ions, combined antibacterial treatments with gallium and non-antibiotic antibacterial agents has also been explored. Akhtar et al. (2020) prepared a gallium-chitosan complex and performed *in vitro* experiments that showed that the antibacterial rate of the complex against *E. coli* was above 90% compared with that of single chitosan. Qiao et al. (2021b) developed a triple combination strategy, as follows: 1) cationic guanidine moieties and short alkyl quaternary ammonium salts were chosen to destroy the bacterial membrane, 2) deferoxamine (DFO)-gallium conjugated to the above copolymer (namely the pGQ-DG complex) was used to disrupt iron metabolism, and 3) vancomycin (VAN) was finally added. The study demonstrated that the fractional inhibitory concentration index (FICI) of sub-MIC concentrations of pGQ-DG combined with VAN was 0.31 in *P. aeruginosa*, confirming their synergy—when FICI values are lower than 0.5, there is a synergistic effect between the drugs tested (Yoon et al., 2006). However, no obvious synergistic effects were observed in *E. coli* between VAN and pGQ-DG. This indicates that the synergistic antibacterial effect between drugs is not absolute and that the synergistic effect will be either enhanced or weakened depending on the bacterial species. It is critical to find optimal synergistic antibacterial formulations for specific pathogens. *In vivo* experiments demonstrated that the three-drug combination had a strong synergistic effect and could achieve the same effect as colistin in the elimination of *P. aeruginosa*, simultaneously accelerating the healing of infected wounds.

Several studies investigated the antimicrobial synergies between different metal ions, and fractional inhibitory concentration and fractional bactericidal concentration assays were used to evaluate synergistic intensity (Yoon et al., 2006; Aziz, 2019; Vaidya et al., 2019; Pormohammad & Turner, 2020). The results showed that silver ions, zinc ions, Cd, Se, and Ga had good synergistic effects. According to Morales-de-Echegaray et al. (2020), the antibacterial activity of gallium-substituted hemoglobin combined with Ag nanoparticles showed multiple amplifications. In addition, antibacterial photodynamic treatment has also been used in combination with gallium to treat bacterial infections. In several studies, gallium-porphyrin, gallium-substituted hemoglobin, phthalocyanine, indocyanine green (ICG), and hollow titanium dioxide nanotubes were used as photosensitizers to couple with gallium (Morales-de-Echegaray et al., 2020; Shisaka et al., 2019; Xie et al., 2021; Yang et al., 2020). Under near-infrared light irradiation, photosensitizers catalyze the production of reactive oxygen substances such as singlet oxygen which has membrane permeability and can cause irreversible oxidative damage to cell membranes, DNA, and lipids (Imlay, 2013). This synergistic mechanism may occur because gallium affects the activity of bacterial antioxidant enzymes by interfering with their iron metabolism, increasing the sensitivity of bacteria to oxidative stress; thus, the reactive oxygen species produced by the action of photosensitizers may promote the killing of bacteria.

Similarly, Zemke et al. (2020) combined nitrates and gallium, where nitrates served as nitric oxide donors—a type of reactive oxygen species—, to induce antibacterial activity against *P. aeruginosa* under both aerobic and anaerobic conditions (Figures 6B,C). Slate et al. (2021) used the sharp nanomorphology of non-metallic graphene and its derivatives to destroy bacterial cell membranes, they simultaneously used gallium that slowly released from graphene foam to interfere with bacterial iron metabolism, which also had a positive synergistic effect.

## 5 Conclusion and Prospective Views

With the recent development of coordination chemistry, an increasing number of organic ligands have been developed to chelate gallium. Therefore, gallium-based compounds have enhanced solubility and improved bioavailability. The coating of biomaterials responsive to an infected microenvironment can greatly increase the gallium ion concentration at the infected site. In addition, further elucidation of the mechanism of gallium acquisition will provide the basis for targeted antibacterial therapy; simultaneously, the constant research on mesoporous materials, bioceramics, hydrogels, bacterial microenvironment-responsive materials, and coating technologies could provide new options for the modification and loading of gallium-based antibacterial agents to realize the sustained and controllable release of gallium (III) and greatly improve the antibacterial effect of this element.

Furthermore, the combined use of various antibacterial agents results in exciting synergistic antibacterial effects. At present, there is a variety of antibacterial drugs, including antibiotics, chitosan, antibacterial peptides, and metal ions, as well as complex antibacterial treatments, such as photothermal treatment, chemical dynamic treatment, photodynamic treatment, and physical destruction. All these antibacterial drugs and methods could be used in combination with gallium, and their combined antibacterial effects remain to be explored. The continuous optimization of the key factors affecting the antibacterial efficiency of gallium will provide the possibility for gallium to be used in the clinic.

However, gallium, as an antibacterial agent, also faces some challenges. Gallium has no indications for patients with specific pathological conditions. Due to its immunosuppressive effect, long-term use of gallium could lead to decreased immune capacity of the body, which is unfortunate for patients with immune deficiencies. In contrast, the antibacterial activity of gallium might be beneficial for patients with hemochromatosis and thalassemia, which cause pathological iron overload. Considering the susceptibility and severity of an infection in these patients, it would be of great clinical significance to successfully use gallium-based antibacterial agents to treat such infections.

Although gallium ions, as multi-target antimicrobial agents, have been demonstrated to have definite and excellent bactericidal effects against pathogens that are resistant to traditional antibiotics, the potency of gallium-based antimicrobials is relatively lower than that of some existing antibacterial drugs. Gallium maltoate has an MIC of more than 250 μg/ml against *P. aeruginosa*, much higher than some clinical antibiotics, such as murepavadin (MIC = 2 μg/ml) and colistin (MIC = 1 μg/ml) (Ekkelenkamp et al., 2020; Piatek et al., 2020). Combination therapies with gallium-based antibacterial agents are expected to further reduce the concentration of gallium ions, so as to be better applied in the treatment of drug-resistant bacteria in the clinic. In addition, understanding the exact antibacterial mechanism of gallium ions remains a long way off. Although gallium has been demonstrated to play an antibacterial role by interrupting iron metabolism, there have been no reports on the proteins or enzymes on which gallium ions act. The identification of gallium ion targets is of great significance for improving gallium antibacterial effects and tackling drug resistance. More research is proposed to focus on the molecular mechanism of the antibacterial activity of gallium in the future. Finally, in recent years, the successful synthesis of various metal–organic frameworks, covalent-organic frameworks, hydrogen-bonded organic frameworks, and other emerging nanomaterials with ultra-high specific surface areas, satisfactory biocompatibility, biodegradability, and high density with uniformly distributed catalytic active sites has greatly promoted the development of antibacterial agents and enriched antimicrobial strategies. However, their combined use with gallium ions is rarely reported, and we believe that this direction possesses promising research prospects.
